# Thyroid gland rupture caused by blunt trauma to the neck

**DOI:** 10.1186/s13104-016-1932-7

**Published:** 2016-02-19

**Authors:** Hirotaka Hara, Yoshinobu Hirose, Hiroshi Yamashita

**Affiliations:** Department of Otolaryngology, Yamaguchi University Graduate School of Medicine, Ube, Yamaguchi 755-8505 Japan

**Keywords:** Thyroid rupture, Blunt trauma, Neck pain

## Abstract

**Background:**

Thyroid rupture following blunt trauma is extremely rare, and neck pain without swelling may be the only presenting symptom. However, hemorrhage and hematoma subsequently causes severe tracheal compression and respiratory distress.

**Case presentation:**

A 71-year-old Japanese woman visited our emergency room with a complaint of increasing right-sided neck pain at the thyroid cartilage level after she tripped and accidentally hit her neck against a pole 3 h back. On admission, her vital signs were stable. There was no swelling or subcutaneous emphysema. Laryngeal endoscopy revealed mild laryngeal edema, although there was no impairment in vocal fold mobility on either side. Contrast-enhanced computed tomography (CT) revealed rupture of the right lobe of the thyroid gland accompanied by a large hematoma extending from the neck to the mediastinum. Under general anesthesia, the right lobe was resected and the hematoma was evacuated.

**Conclusion:**

Only a few isolated cases of thyroid rupture caused by blunt neck trauma have been reported in patients with normal thyroid glands and neck pain without swelling may be the only presenting symptom. When suspected, CT should be performed to confirm the diagnosis determine the optimal treatment.

## Background

Thyroid rupture following blunt trauma to the neck is extremely rare. Because the thyroid gland is located in the anterior cervix, neck pain without swelling may be the only presenting symptom immediately after neck trauma. However, hemorrhage and hematoma subsequently causes severe tracheal compression and respiratory distress. Here we report the case of a 71-year-old woman whose thyroid ruptured after blunt neck trauma, leading to the formation of a large hematoma extending from the paratracheal region to the upper mediastinum. Successful treatment was achieved with hemithyroidectomy and hematoma removal.

## Case report

**Fig. 1:**
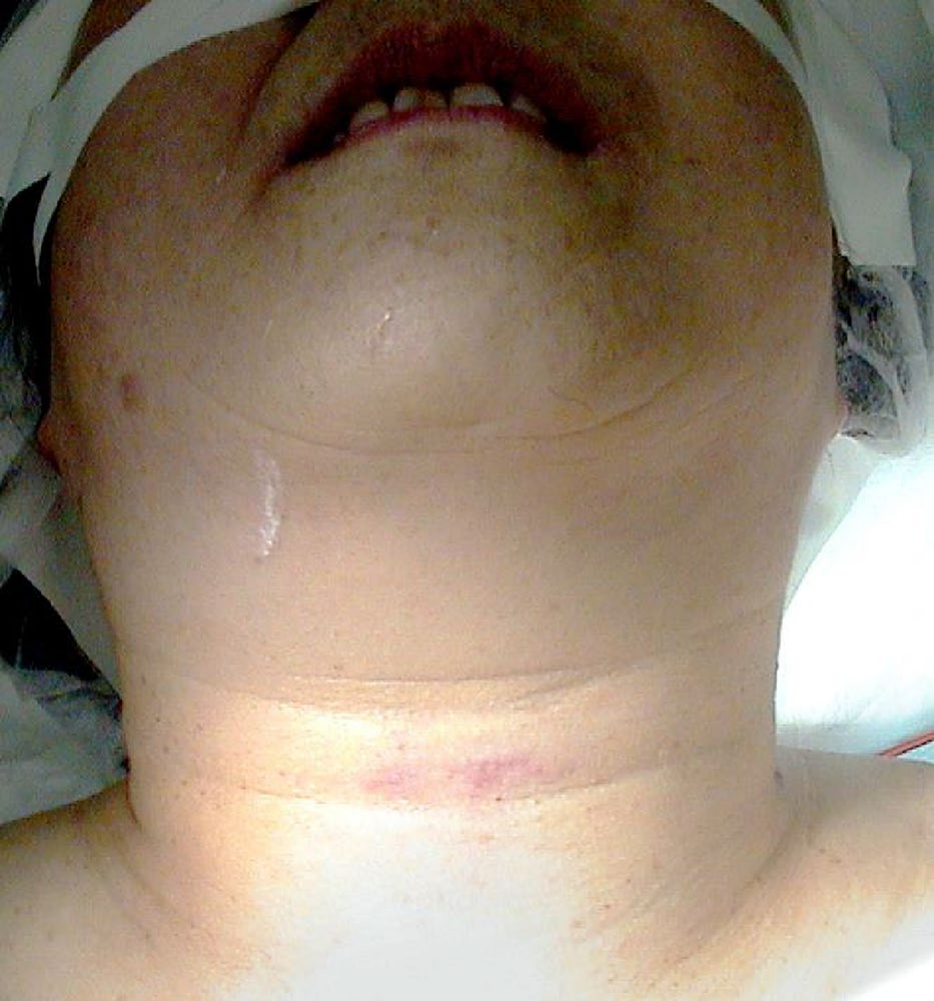
Preoperative image of the patients neck. Erythema can be noted at the trauma site at the level of the thyroid cartilage

A 71-year-old woman presented to our emergency room with a complaint of increasing right-sided neck pain at the thyroid cartilage level after she accidentally tripped and hit her neck against a pole 3 h back. At the time of admission, her vital signs were stable without evidence of respiratory distress, and there was no swelling or subcutaneous emphysema (Fig. 1). Laryngeal endoscopy revealed normal vocal fold mobility and mild edema of the arytenoids.

**Fig. 2:**
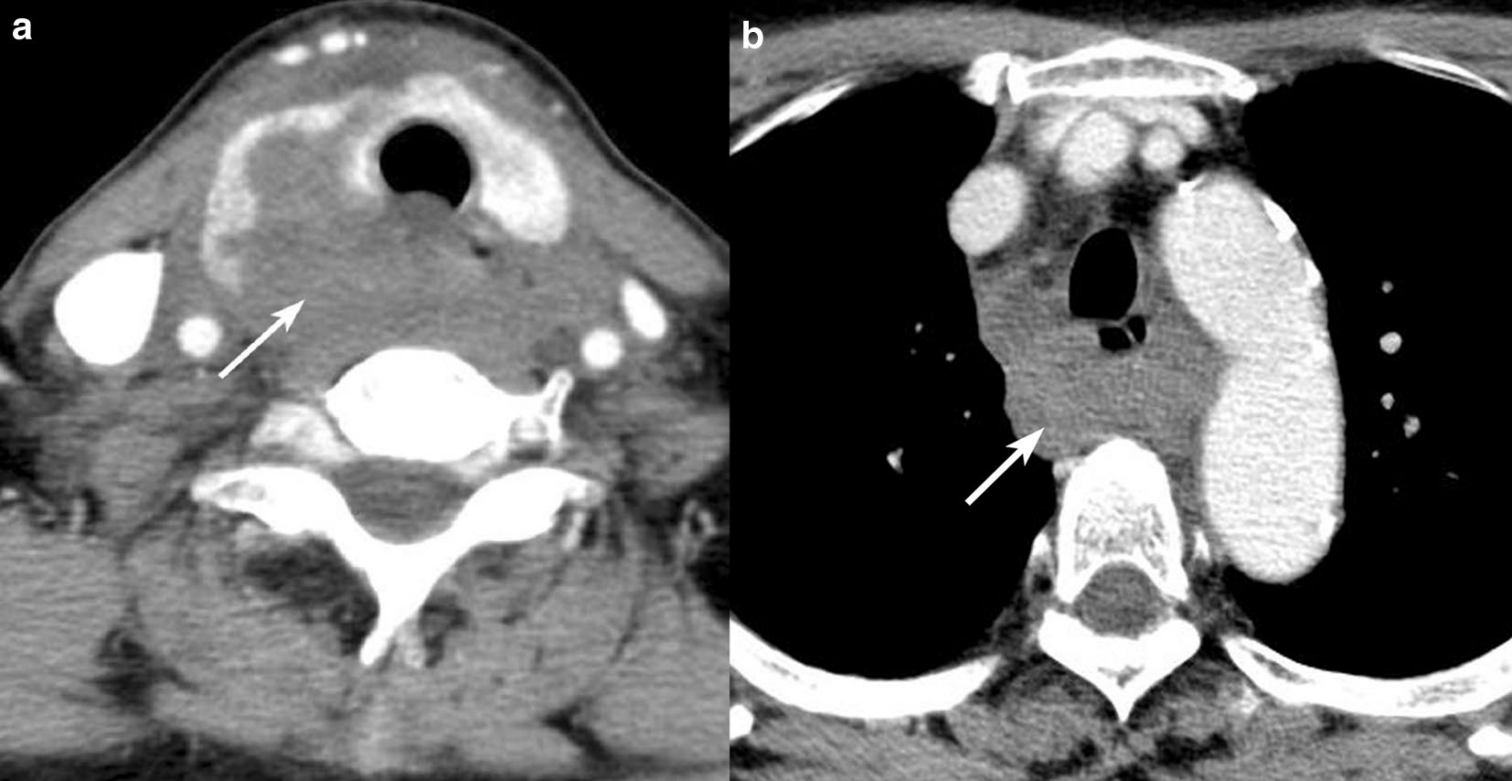
Computed tomography of the neck and chest. Computed tomography showing thyroid rupture, with a large hematoma (*arrow*) extending from the neck (**a**) to the mediastinum (**b**)

**Fig. 3:**
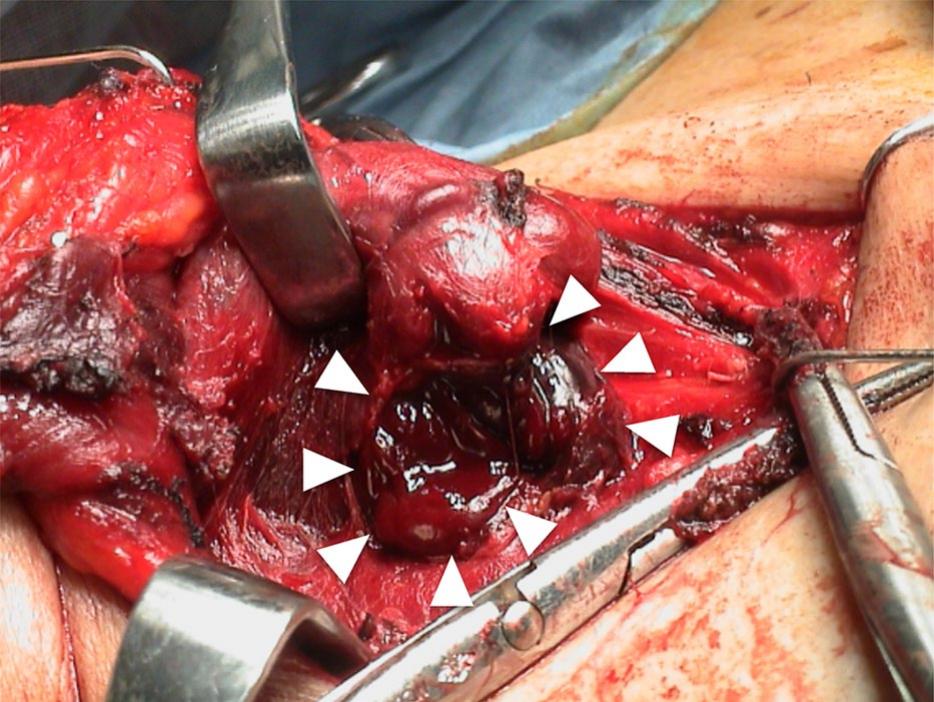
Intraoperative views. The dorsal side of the right lobe of the thyroid gland is lacerated and ruptured. The hematoma (*arrowhead*) is extending along the paratracheal region to the upper mediastinum

**Fig. 4:**
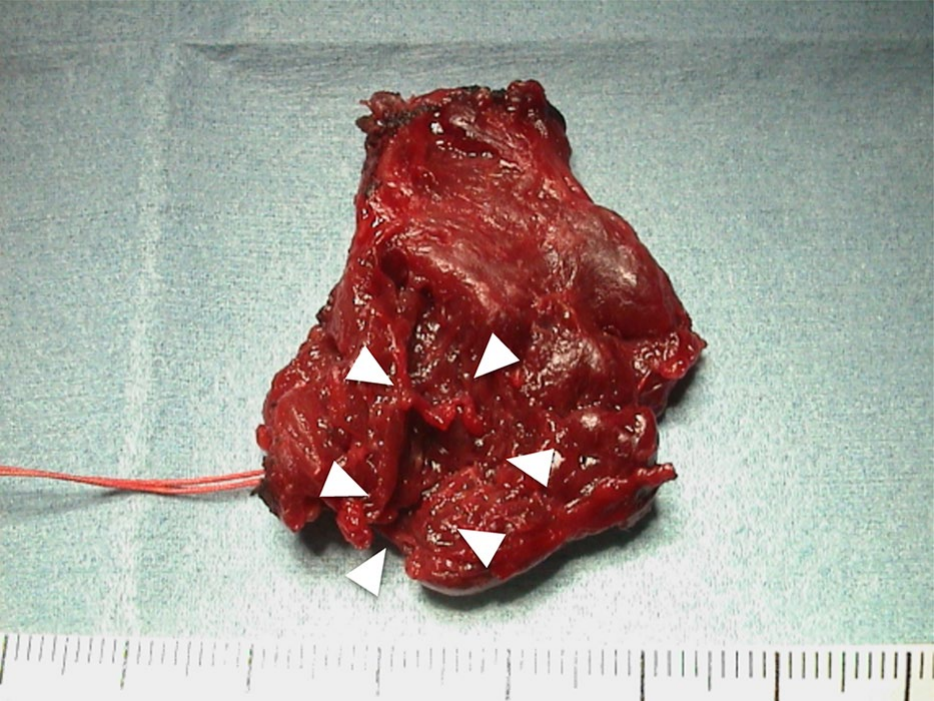
Gross findings of the resected right lobe of the thyroid. Lacerations (*arrowhead*) are evident on the dorsal side of the right lobe of the thyroid gland

Contrast-enhanced computed tomography (CT) revealed rupture of the right lobe of the thyroid gland accompanied by a large hematoma extending from the thyroid to the mediastinum (Fig. 2a, b). There were no cysts or goiter in either thyroid lobe and no fracture of the laryngeal framework, cartilages, and trachea. In view of the patient’s condition and the presence of the large hematoma, neck exploration under general anesthesia was undertaken. During surgery, the dorsal side of the right lobe of the thyroid gland was found to be ruptured with lacerations (Figs. 3, 4). The ruptured right lobe was resected and the hematoma, which extended to the upper mediastinum, was evacuated. Pathological examination of the resected lobe revealed otherwise normal anatomy, with no evidence of goiter or cyst.

The patient required postoperative intubation for 24 h because of laryngeal edema, but she subsequently made a full and uneventful recovery.

## Discussion

Thyroid injury secondary to blunt neck trauma is uncommon and mostly occurs in patients with preexisting goiters [1, 2]. Only a few isolated cases of thyroid rupture caused by blunt neck trauma have been reported in patients with normal thyroid glands [3–7]. Common causes of thyroid injury include traffic accidents, particularly with motorcycles or bicycles, and falls or slips. Although extremely rare, spontaneous thyroid hemorrhage following the muscular effort of heavy lifting has also been reported [2–4, 8–14]. Although rare, thyroid injury secondary to blunt neck trauma is clinically significant because hematomas can present inconspicuously and cause respiratory distress and serious complications, particularly if they are large and extending to the mediastinum.

To confirm the diagnosis of thyroid rupture, ultrasound (US) and computed tomography are essential [3, 11]. CT reportedly exhibits high sensitivity in diagnosing tracheal ruptures in patients with blunt neck trauma and is the initial diagnostic modality of choice in stable patients [3, 4] because it also permits the evaluation of other structures in the anterior cervix. Doppler-mode US can be useful for assessing thyroid lacerations and hematomas and is particularly useful in the emergency room prior to emergency CT. In our patient, we used both US and CT because the nature of the injury necessitated a detailed assessment of other vital structures, including the laryngeal framework cartilages, trachea, and large vessels.

To minimize the risk of breathing distress, laryngeal or bronchial fibroscopy should be performed to assess laryngeal edema and evaluate vocal fold mobility. Although our patient did not complain of dyspnea on admission, laryngeal fibroscopy revealed arytenoid edema.

According to the literature, the management of this type of injury varies. The majority of patients require close observation because of the potential seriousness of any complications, and several patients have reportedly undergone hemi or total thyroidectomy [1–18]. Conservative or surgical treatment should be determined on the basis of CT findings and vital signs. Securing the airway when necessary should be the priority [15]. The thyroid gland has a rich blood supply that can easily result in an expanding hematoma capable of causing airway compromise. If a hematoma is present, surgical intervention may be necessary, and if not, close monitoring and conservative treatment may be appropriate [16]. Given the rarity of the condition, it is difficult to recommend duration of monitoring. However, Chartier and Turner reported a 75 year-old lady who developed respiratory distress from a hematoma as late as 33 h after initial trauma [17].

The primary reason for surgical intervention in our patient was evacuation of the rapidly extending hematoma. The retropharyngeal prevertebral space is an important pathway from the neck to the mediastinum, and deep neck infections usually follow this route. Airway distress can occur in patients with massive hemorrhage from the thyroid or a branch of the internal jugular vein. Because there was a massive hematoma extending to the upper mediastinum, with the risk of severe complications, we opted for open surgery and evacuation of the hematoma.

Isolated thyroid rupture is very rare. Sow et al. [18] reported a case of with fracture of the thyroid cartilage, mandible, and both first ribs. There are few case reports of blunt neck trauma causing thyroid rupture in elderly patients. In Japan, many elderly patients with locomotive disorders are at risk of falls and neck trauma. Furthermore, many elderly patients may be on anticoagulant therapy for the prevention of infarction or atherosclerosis, thus increasing their risk of hemorrhage. Fortunately, our patient was not on any anticoagulant therapy; nevertheless, physicians should bear this in mind when managing an elderly patient after a fall.

## Conclusions

We described a rare case of thyroid rupture following blunt neck trauma. When suspected, CT should be performed to confirm the diagnosis, and if there is evidence of a hematoma, surgical intervention may be necessary. For uncomplicated cases, conservative treatment and monitoring is recommended.

## Ethics

Permission for publication of this case was obtained by Yamaguchi University Hospital Institutional Review Board.

## Consent

Written informed consent was obtained from the patient for publication of this case report and any accompanying images.

## Abbreviations

CT: computed tomography
US: ultrasound
